# DOTS improves treatment outcomes and service coverage for tuberculosis in South Ethiopia: a retrospective trend analysis

**DOI:** 10.1186/1471-2458-5-62

**Published:** 2005-06-06

**Authors:** Estifanos B Shargie, Bernt Lindtjørn

**Affiliations:** 1 University of Bergen Centre for International Health, Armauer Hansens Hus, N-5021 Bergen, Norway; 2 Southern Nations, Nationalities and Peoples' Regional State Health Bureau, P. O. Box 149, Awassa, Ethiopia

## Abstract

**Background:**

DOTS as a strategy was introduced to the tuberculosis control programme in Southern region of Ethiopia in 1996. The impact of the programme on treatment outcomes and the trend in the service coverage for tuberculosis has not been assessed ever since. The aim of the study was to assess trends in the expansion of DOTS and treatment outcomes for tuberculosis in Hadiya zone in Southern Ethiopia.

**Methods:**

19,971 tuberculosis patients registered for treatment in 41 treatment centres in Hadiya zone between 1994 and 2001 were included in the study. The data were collected from the unit tuberculosis registers. For each patient, we recorded information on demographic characteristics, treatment centre, year of treatment, disease category, treatment given, follow-up and treatment outcomes. We also checked the year when DOTS was introduced to the treatment centre.

**Results:**

Population coverage by DOTS reached 75% in 2001, and the proportion of patients treated with short course chemotherapy increased from 7% in 1994 to 97% in 2001. Treatment success for smear-positive tuberculosis rose from 38% to 73% in 2000, default rate declined from 38% to 18%, and treatment failure declined from 5% to 1%. Being female patient, age 15–24 years, smear positive pulmonary tuberculosis, treatment with short course chemotherapy, and treatment at peripheral centres were associated with higher treatment success and lower defaulter rates.

**Conclusion:**

The introduction and expansion of DOTS in Hadiya has led to a significant increase in treatment success and decrease in default and failure rates. The smaller institutions exhibited better treatment outcomes compared to the larger ones including the zonal hospital. We identified many patients with missing information in the unit registers and this issue needs to be addressed. Further studies are recommended to see the impact of the programme on the prevalence and incidence of tuberculosis.

## Background

Ethiopia stands among the world's top 22 tuberculosis (TB) high-burden countries, with an estimated annual incidence of 250 TB cases/10^5 ^population [1,2]. In the Southern Ethiopia Regional State (SNNPRS), TB is among the leading causes for sickness and death [3]. As in many other resource-constrained settings, treatment outcomes for tuberculosis have not been satisfactory [4], mainly due to poor treatment compliance and low coverage of short course chemotherapy (SCC). Delays in the diagnosis and treatment initiation, the devastating HIV/AIDS epidemic and the potential threat of anti-tuberculosis drug resistance represent serious threats to the TB control effort in the region. The HIV co-infection among TB patients in the region is estimated at 19% [5].

Directly Observed Treatment, Short-course (DOTS) was introduced in the region in 1996. However, the impact of DOTS and its effectiveness in the regional context has not been assessed yet. Global reviews [6] and reports from Asian, African and East European countries [7-9] have favourable implications for the DOTS strategy. On the contrary, some randomised controlled studies have failed to establish the superiority of the most notable component of this strategy, direct observation of treatment, over the conventional non-observed treatment in improving treatment outcomes [10,11]. Nevertheless, DOTS is widely accepted and practised, with some TB high-burden countries achieving almost full coverage of their populations [2]. DOTS is equally effective in curing TB patients with HIV co-infection [9,12] and hence, its importance in the era of the HIV/AIDS epidemic.

With this study, we aimed to assess the results and trends in the expansion of DOTS in Hadiya Zone, Southern Ethiopia.

## Methods

### Study setting

**Figure 1:**
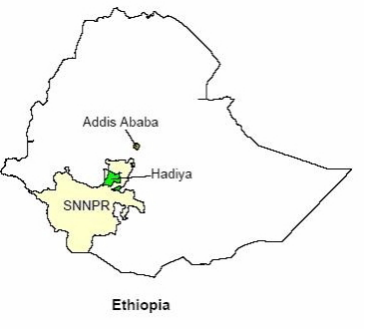
**Map of Ethiopia with the study area highlighted**. Ethiopia is administratively divided into nine regional states and two city administrations, and the Southern Nations, Nationalities and Peoples' Regional State (SNNPRS) accounts for one-fifth (13 million) of the total population of the country. The study area (Hadiya zone) has got a population of 1.2 million.

We did the study in Hadiya zone, Southern Ethiopia (see map in Figure 1), with a population of 1.2 million [13,14]. In addition to fifteen diagnostic centres, five health centres and 21 health stations provide treatment for TB patients referred or transferred from the diagnostic centres, thus making the number of treatment centres 41. All treatment units have standard Unit Registers from the National Tuberculosis and Leprosy Control Programme (TLCP). TB drugs used in a combination of two or more in the region included isoniazid (H), rifampicin (R), pyrazinamide (Z), ethambutol (E), streptomycin (S) and thioacetazone (T).

The TB and Leprosy control programs were run as two separate vertical programs funded and managed mainly by the All African Leprosy and Rehabilitation Training Centre (ALERT) until the end of 1996 when the National and Regional TLCP took over the responsibility. The zonal TLCP gets administrative and technical support from the regional TLCP, and monitors the activities in the diagnostic and treatment centres through its seven woreda (district) offices. The woreda health offices have standard district TB registers from the National TLCP, and are responsible for the distribution of drugs and reagents, supervision of day to day activities in the treatment units, collection of slides for quality control and record keeping. Quarterly reports on cases detected and treatment outcomes from the woredas are gathered at the zonal level and sent to the regional TLCP.

In 1994, only Hossana Hospital provided unobserved SCC for critically sick smear-positive pulmonary TB (PTB+), miliary TB and tuberculosis meningitis cases. DOTS was initiated in 1996 in two health facilities and gradually expanded, first to the health centres in all woredas and then to the health stations. Details of treatment regimens for various categories of patients under DOTS are given in the NTLCP manual [15]. In brief, all new patients were treated with SCC (two months on RHZ +/- E or S, followed by 6 months on EH or RH), and were required to take their medications under direct supervision by the health workers at least during the intensive phase of treatment. During this phase, only critically ill patients were hospitalised, while the rest received treatment on ambulatory basis. Re-treatment cases were treated with SERHZ for 2 months, ERHZ for 1 month and ERH for 5 months. In non-DOTS areas treatment with SCC was limited to critically sick PTB+, TB meningitis and miliary TB cases while others received LCC (two months on EH +/-S, followed by 10 months on EH; TH had also been used as an alternative to EH until its recent withdrawal).

### Design and data collection

This is a retrospective trend analysis. The Unit Registers reviewed contain basic information such as patient's age, sex, address, category, TB type, drug regimen, date treatment started, treatment follow-up, follow-up sputum result and treatment outcomes.

We visited all 41 TB treatment centres during July-November 2002. We checked when DOTS was initiated in each health institution and looked for the availability of TB drugs and reagents during the time of visit. We then reviewed the Unit Registers and entered the data to the computer. At three peripheral treatment centres where the unit registers could not be found, the respective district TB registers were reviewed. For each TB case, we copied the data from the registers to a computer program, SPSS for windows [16], according to the standard definitions of the National TLCP [15] on disease classification, patient categories, treatment regimens and treatment outcomes. To ensure the quality of data entered into the computer database, two people independently cross-checked each entry.

### Data analysis and Statistics

The data were analysed using SPSS for Windows version 11.0 [16]. For categorical data, we used proportions with 95% confidence intervals, Odds ratio and Chi-square test to compare different groups. Multivariate analysis using logistic regression model was used to analyse the association between treatment outcomes and potential predictor variables. We set the level of statistical significance at 5%.

Treatment outcomes were analysed for the years 1994–2000 because a considerable proportion of patients registered during 2001 were still on treatment during the time of data collection. (The Ethiopian fiscal year goes from July to June. Fiscal year 2001, for example, goes from July 2001 through June 2002, and our study was commenced in July 2002). Patients with unrecorded treatment outcome were analysed as defaulters.

## Results

### Patient registration and case notification

A total of 19971 tuberculosis patients, 11138 (55.8%) males and 8819 (44.2%) females were registered between 1994 and 2001 with the mean (SD) age of 25.6 (13.2) years. Forty-six percent (n = 9232) of the patients were PTB+ cases. Overall, 18687 (93.6%) patients were registered as new cases, 558 (2.8%) as transferred in, 273 (1.4%) as return after default, 142 (0.7%) as failure and 139 (0.7%) as relapse cases whereas patient category was not recorded for 172 (0.9%) cases. Table 1 shows the general characteristics of the patients.

**Table 1:** General Characteristics of the study subjects (n = 19971), 1994–2001

**Characteristics**	**Number**	**Percent**
***Age group (years)***		
0–14	3356	16.8
15–24	6262	31.4
25–34	5366	26.9
35–44	2706	13.5
45–54	1248	6.2
55–64	501	2.5
≥ 65	220	1.1
Unknown	312	1.6
***Sex***		
Male	11138	55.8
Female	8819	44.2
Not mentioned	14	0.1
***Patient Category***		
New	18687	93.6
Transferred-in	558	2.8
Return after default	273	1.4
Failure	142	0.7
Relapse	139	0.6
Unknown	172	0.9
***TB Classification***		
Pulmonary positive	9232	46.2
Pulmonary negative	4225	21.2
Extra-pulmonary	6453	32.3
Unknown	61	0.3
***Treatment Centre***		
Hossana Hospital	5362	26.8
Lemmo district health facilities	4011	20.1
Shashogo district health facilities	1208	6.0
Misha district health facilities	2660	13.3
Gibe district health facilities	1984	9.9
Soro district health facilities	2313	11.6
Duna district health facilities	216	1.1
Badewacho district health facilities	2217	11.1

The proportion of women among TB patients registered for treatment remained at the range of 40–45% across the years and at the range of 43–47% across the age groups below 45. However, among patients older than 45 years, the proportion of women was significantly lower (31%; 95%CI 29–33) compared to those below 45 (46%; 95%CI 45–47).

Among new cases, 46% (n = 8557) were PTB+ patients. Except for the years 1997 (38%; 95%CI 36–40) and 2000 (56%; 95%CI 54–58), this proportion showed little variations in the range of 40–50%. Table 2 shows a trend in the case notification of PTB+ over the study period. The proportion of expected PTB+ incident cases notified increased from 45% in 1994 to 116% in 1999, and declined to 67% in 2001.

**Table 2:** Trends in case notification of smear-positive pulmonary TB, Hadiya Zone, 1994–2001

Year	Zonal Population*	New PTB+ cases Reported (n = 8558)	Case Notification/ 10^5 ^persons/year	Proportion of estimated PTB+ incident cases notified, %**
1994	1004000	512	49	45.2
1995	1070160	1025	96	87.9
1996	1101195	1162	106	96.8
1997	1133129	946	84	76.6
1998	1165990	1277	110	100.5
1999	1199804	1510	126	115.5
2000	1234598	1267	103	94.2
2001^§^	1174118	858	73	67.0

*Projected from the 1994 Population and Housing Census, CSA [13]

**Estimated Incidence of PTB+ = 109/10^5 ^persons/year based on the WHO estimates of the Global Burden of Tuberculosis for 1997 [1]

^§ ^Population adjusted as an area with about 70–80 thousand population was taken away from Hadiya to the neighbouring Silti zone between years 2000 and 2001

PTB+: smear-positive pulmonary tuberculosis

### Treatment given

Forty percent (n = 7919) of patients received SCC. Treatment regimen was not recorded for 981 (5%) cases. Table 3 presents trend in treatment regimens across the years by TB classification. The proportion of patients treated with SCC increased from 7% in 1994 to 58% in 1999, and 97% in 2001 (χ^2^_trend_, p < 0.001).

**Table 3:** Trend in treatment regimens for the different categories of patients (n = 19970).*

**Category**	**1994 n (%)**	**1995 n (%)**	**1996 n (%)**	**1997 n (%)**	**1998 n (%)**	**1999 n (%)**	**2000 n (%)**	**2001 n (%)**
**All TB cases**	1106	2201	2895	2742	3388	3253	2435	1950
SCC	73 (7)	311 (14)	288(10)	471 (17)	927 (27)	1881 (58)	2085 (86)	1883 (97)
LCC	1011 (91)	1684 (77)	2360 (82)	2097 (77)	2292 (68)	1302 (40)	304 (12)	20 (1)
No record	22 (2)	206 (9)	247 (8)	174 (6)	169 (5)	70 (2)	46 (2)	47 (2)
								
**New PTB+**	512	1025	1162	946	1277	1510	1267	858
SCC	9 (2)	111 (11)	94 (8)	232 (24)	537 (42)	1170 (78)	1187 (94)	837 (97)
LCC	496 (97)	780 (76)	938 (81)	611 (65)	660 (52)	321 (21)	68 (5)	7 (1)
No record	7 (1)	134 (13)	130 (11)	103 (11)	80 (6)	19(1)	12 (1)	14 (2)
								
**New PTB-**	258	385	402	382	807	772	487	393
SCC	12 (5)	66 (17)	90 (22)	109 (28)	225 (28)	395 (51)	402 (83)	379 (96)
LCC	242 (94)	286 (74)	248 (62)	251 (66)	543 (67)	362 (47)	73 (15)	-
No record	4 (1)	33 (9)	64 (16)	22 (6)	39 (5)	15 (2)	12 (2)	14 (4)
								
**New EPTB**	257	668	1177	1176	1124	721	528	570
SCC	19 (8)	73 (11)	57 (5)	70 (6)	97 (9)	197 (27)	385 (73)	559 (98)
LCC	232 (90)	565 (85)	1081 (92)	1067 (91)	993 (88)	513 (71)	138 (26)	6 (1)
No record	6 (2)	30 (4)	39 (3)	39 (3)	34 (3)	11 (2)	5 (1)	5 (1)
								
**Relapse**	8	13	21	29	15	19	19	15
SCC	2 (25)	10 (77)	12 (57)	21 (72)	13 (87)	17 (90)	19 (100)	14 (93)
LCC	4 (50)	2 (15)	9 (43)	6 (21)	2 (13)	2 (10)	-	-
No record	2 (2)	1 (8)	-	2 (7)	-	-	-	1 (7)
								
**After default**	35	56	27	45	33	25	21	31
SCC	5 (14)	14 (25)	15 (56)	20 (45)	15 (46)	21 (84)	17 (81)	30 (97)
LCC	30 (86)	39 (70)	11(41)	23 (51)	18 (54)	4 (16)	4 (19)	-
No record	-	3 (5)	1 (3)	2 (4)	-	-	-	1 (3)
								
**After failure**	28	38	17	17	13	19	8	2
SCC	26 (93)	35 (92)	15 (88)	13 (77)	11 (85)	14 (74)	8 (100)	2 (100)
LCC	2 (7)	3 (8)	2 (12)	4 (23)	2 (15)	5 (26)	-	-
No record	-	-	-	-	-	-	-	-
								
**Transferred in**	6	3	65	131	89	120	86	57
SCC	-	-	3 (5)	3 (2)	22 (25)	43 (36)	53 (62)	38 (67)
LCC	5 (83)	2 (67)	55 (84)	124 (95)	58 (65)	57 (47)	19 (22)	7 (12)
No record	1 (17)	1 (33)	7 (11)	4 (3)	9 (10)	20 (17)	14 (16)	12 (21)
								
**Missing info**	2	13	17	10	20	46	13	29
SCC	-	2 (15)	1 (5)	3 (30)	3 (15)	11 (24)	9 (69)	29 (100)
LCC	-	7 (54)	12 (71)	6 (60)	11 (55)	30 (65)	2 (16)	-
No record	2	4 (31)	4 (24)	1 (10)	6 (30)	5 (11)	2 (15)	-

* Percentages are out of the total cases registered in the same category during the year.

PTB+ = smear-positive pulmonary tuberculosis; PTB- = smear-negative pulmonary tuberculosis; EPTB = extra-pulmonary tuberculosis

SCC = short course chemotherapy (2 months on RHZ+/- E or S, followed by 6 months on EH or RH)

LCC = long course chemotherapy (2 months on EH +/- S, followed by 10 months on EH; TH had also been used as an alternative to EH until its recent withdrawal).

R = Rifampicin,; H = Isoniazid; Z = Pyrazinamide; E = Ethambutol; S = Streptomycin; T = Thioacetazone.

During 1994–1997, more PTB- patents received SCC compared to PTB+ patients (19% vs. 12%; p < 0.05) and EPTB patients (19% vs. 7%; p < 0.05) among new cases of TB (Table 3). This trend was reversed after 1997 when more PTB+ patients (76%) were put on SCC, followed by PTB- (57%) and EPTB (42%). There was no significant difference in the treatment regimens across the age groups and between the two sexes.

### Follow-up

Of the 8557 new PTB+ cases, sputum examination was repeated at the end of two months treatment for 60% (n = 5112), and 7.8% (n = 401) remained positive for AFB. At the end of five months treatment, 2747 (32%) patients had their sputum examined for AFB, and 78 (2.8%) remained smear-positive. 1823 (21%) patients had their sputum examined for AFB at (a month prior to) treatment completion, and 1.4% (n = 26) remained smear-positive. There has been a continuous and significant decline over time in the sputum positivity at two months irrespective of treatment regimen (Table 4).

**Table 4:** Trends in the follow-up smear results for new smear positive pulmonary tuberculosis

	**2^nd ^month**		**5^th ^month**		End of treatment	
	
	*Examined for AFB (% total)*	*sm+*	*Examined for AFB (% total)*	*Sm+*	*Examined for AFB (% total)*	*sm+*
**Cases on SCC**						
1994	6 (67)	0	5 (56)	0	3 (33)	0
1995	79 (71)	18 (23%)	53 (48)	4 (8%)	33 (30)	0
1996	68 (72)	15 (22%)	38 (40)	2 (5%)	25 (27)	0
1997	155 (67)	14 (9%)	94 (41)	0	74 (32)	2 (3%)
1998	342 (64)	24 (7%)	183 (34)	0	140 (26)	0
1999	824 (70)	52 (6%)	411 (35)	7 (2%)	365 (31)	2 (0.5%)
2000	817 (69)	43 (5%)	439 (37)	9(2%)	316 (27)	2 (1%)
2001^§^	527 (63)	13 (3%)	171 (20)	1 (0.6%)	121 (14)	0
Cases on LCC						
1994	330 (66)	45 (14%)	154 (31)	19 (12%)	86 (18)	5 (6%)
1995	508 (65)	75 (15%)	354 (45)	15 (4%)	179 (23)	4 (2%)
1996	564 (60)	59 (11%)	385 (41)	7 (2%)	223 (24)	4 (2%)
1997	302 (49)	15 (5%)	188 (31)	5 (3%)	86 (14)	3 (4%)
1998	263 (40)	19 (7%)	127 (19)	7 (6%)	80 (12)	2 (3%)
1999	182 (57)	7 (4%)	86 (27)	1 (1%)	68 (21)	2 (3%)
2000	46 (68)	1 (2%)	9 (13)	0	4 (6)	0
2001^§^	2 (29)	1	2 (29)	0	1 (14)	0
**Unknown regimen**						
1994	0	0	2 (29)	1	2 (29)	0
1995	9 (7)	0	4 (3)	0	3 (2)	0
1996	25 (19)	0	9 (7)	0	3 (2)	0
1997	23 (22)	0	11 (11)	0	3 (3)	0
1998	27 (34)	0	19 (24)	0	5 (6)	0
1999	4 (21)	0	1 (5)	0	1 (5)	0
2000	4 (33)	0	1 (8)	0	0	0
2001^§^	5 (36)	0	17)	0	2 (14)	0

sm+ = sputum smear positive for acid-fast bacilli out of examined.

SCC = short course chemotherapy; LCC = long course chemotherapy; AFB = acid-fast bacilli.

^§^Follow-up smears for the 5^th ^month and end of treatment were not complete for the year 2001 as a considerable number of patients were still on treatment during the time of data collection.

Sputum positivity at the completion of fifth month treatment remained at the range of 0–2% for those on SCC, with the exception of 1995 and 1996 that exhibited 8% and 5% respectively. For those on LCC, smear positivity at 5^th ^month decreased from 12% in 1994 to 1% in 1999. Overall, patients on LCC contributed more to the 5^th ^month and end of treatment smear-positivity (treatment failure), than those on SCC (p < 0.05; table 4).

### Treatment results

We analysed treatment outcomes for the years 1994–2000 and, as a result, we evaluated 16943 (85% of the total) cases. Of these, 8268 (49%) successfully completed treatment, 3151 (18.6%) defaulted, 110(0.6%) had treatment failure, 446 (2.6%) died and 2000 (10%) were transferred out, while 2968 (17.5%) had no record of treatment outcomes. Assuming that patients with unrecorded treatment outcome had all defaulted from treatment, a total of 6119 (36.1%) patients defaulted.

Among new PTB+ (and all TB) patients, treatment success was higher among patients on SCC, while default and failure rates were higher among those on LCC (Table 5). Death in all categories was higher among those on SCC. Among new cases on SCC, PTB+ cases had 1.8 (65% vs.37%) and 1.4 (65% vs. 48%) times higher rate of treatment success compared to PTB- and EPTB cases, respectively (p < 0.001). Female PTB+ patients had significantly higher treatment success (58% vs. 54%; p = 0.001) and lower defaulter rate (26% vs. 30%; p < 0.001) than males.

**Table 5:** Treatment outcomes for different categories of patients on short and long course regimens (n = 16943)

	**New cases**						
					
	**PTB+ (%)**	**PTB- (%)**	**EPTB (%)**	**Relapse (%)**	**After default (%)**	**After failure (%)**	**Transferred in (%)**
**Patients on SCC**							
Treatment success	2166 (65)	475 (37)	436 (48)	62 (66)	49 (46)	65 (53)	79 (64)
Default*	726 (22)	483 (37)	320 (36)	13 (14)	45 (42)	31 (25)	33 (26)
Failure	25 (0.7)	-	-	2 (2)	1 (1)	6 (5)	3 (2)
Death	117 (3.5)	72 (5)	24 (3)	11 (12)	5 (5)	2 (1)	2 (2)
Transferred out	306 (9)	269 (21)	118 (13)	6 (6)	7 (7)	18 (15)	7 (6)
							
**Patients on LCC**							
Treatment success	1986 (51)	772 (39)	1932 (42)	14 (56)	32 (25)	11 (61)	189 (59)
Default*	1365 (35)	972 (48)	1922 (42)	8 (33)	79 (61)	2 (11)	24 (8)
Failure	66 (2)	-	-	1 (4)	2 (2)	3 (17)	1 (0.3)
Death	107 (3)	57 (3)	37 (1)	2 (8)	2 (2)	1 (5)	7 (2)
Transferred out	350 (9)	204 (10)	697 (15)	-	14 (11)	1 (6)	3 (1)

* Patients with missing treatment outcome information were assumed to have defaulted and analysed as defaulters.

PTB+ = smear positive pulmonary tuberculosis; PTB- = smear negative pulmonary tuberculosis; EPTB = extra-pulmonary tuberculosis

SCC = short course chemotherapy (2 months on RHZ+/- E or S, followed by 6 months on EH or RH)

LCC = long course chemotherapy (2 months on EH +/-S, followed by 10 months on EH; TH had also been used as an alternative to EH until its recent withdrawal).

R = Rifampicin,; H = Isoniazid; Z = Pyrazinamide; E = Ethambutol; S = Streptomycin; T = Thioacetazone.

Patients registered as "return after default", had significantly lower treatment success compared to new cases, both for all TB (34% vs. 49%; p < 0.001), and for PTB+ (34% vs. 58%; p < 0.001). Furthermore, return after default cases were more likely to default again compared to new cases among PTB+ patients (53% vs. 29%; p < 0.001) and all TB patients (53% vs. 36%; p < 0.001). Treatment failure was five times higher among cases that received re-treatment for previous failure compared to new cases of PTB+ (6.4% vs. 1.2%; p < 0.001); however, both groups had comparable treatment success, mainly due to a lower defaulter rate among failure cases (24% vs. 29%).

Patients for whom follow-up sputum smear remained positive at the second month during treatment had significantly lower treatment success than those with negative smear result (53% vs. 74%; p < 0.001), mainly as a result of higher defaulter (24% vs. 18%) and failure (10% vs. 1%) rates among the former group.

Table 6 presents treatment outcomes for new cases of TB across the years. A steady rise in treatment success and decline in defaulter rate was noticed particularly among patients on SCC. Treatment failure showed a remarkable decline in both SCC and LCC groups. Death rate did not show much variation across the years.

**Table 6:** Treatment outcomes across the years for new cases of tuberculosis

	**1994 n (%)**	**1995 n (%)**	**1996 n (%)**	**1997 n (%)**	**1998 n (%)**	**1999 n (%)**	**2000 n (%)**
PTB+ on SCC							
Treatment success	5 (42)	40 (34)	45 (38)	122 (47)	338 (60)	811 (69)	805 (74)
Default*	5 (42)	61 (51)	50 (42)	77 (30)	114 (20)	225 (19)	194 (17)
Failure	-	3 (3)	2 (2)	2 (1)	-	8 (1)	10 (1)
Death	-	4 (3)	2 (2)	10 (4)	24 (4)	44 (4)	33 (3)
Transferred out	2 (17)	11 (9)	20 (17)	46 (18)	89 (16)	88 (7)	50 (5)
							
PTB+ on LCC							
Treatment success	188 (38)	384 (48)	564 (56)	271 (45)	308 (52)	231 (72)	40 (68)
Default*	190 (38)	298 (38)	304 (30)	246 (40)	240 (40)	72 (22)	15 (25)
Failure	23 (5)	18 (2)	8 (1)	8 (1)	6 (1)	3 (1)	-
Death	17 (3)	21 (3)	18 (2)	20 (3)	19 (3)	9 (3)	3 (5)
Transferred out	78 (16)	71 (9)	107 (11)	63 (10)	24 (4)	6 (2)	1 (2)
							
PTB- on SCC							
Treatment success	1 (8)	15 (23)	18 (20)	32 (29)	67 (30)	153 (39)	189 (47)
Default*	8 (67)	40 (60)	67 (74)	55 (50)	85 (38)	116 (29)	112 (28)
Death	1 (8)	6 (9)	2 (2)	10 (9)	8 (4)	25 (6)	20 (5)
Transferred out	2 (17)	5 (8)	3 (3)	12 (11)	65 (29)	101 (26)	81 (20)
							
PTB- on LCC							
Treatment success	107 (44)	88 (31)	84 (34)	78 (31)	199 (37)	182 (50)	34 (47)
Default*	101 (42)	163 (57)	127 (51)	141 (57)	280 (51)	135 (37)	25 (34)
Death	4 (2)	5 (2)	6 (2)	5 (2)	16 (3)	20 (6)	1 (1)
Transferred out	30 (12)	30 (11)	31 (13)	27 (11)	48 (9)	25 (7)	13 (18)
							
EPTB on SCC							
Treatment success	4 (21)	19 (26)	25 (44)	29 (32)	43 (44)	101 (51)	215 (56)
Default*	11 (58)	43 (59)	29 (51)	30 (33)	29 (30)	68 (35)	110 (28)
Death	2 (11)	6 (8)	1 (2)	3 (4)	1 (1)	4 (2)	7 (2)
Transferred out	2 (11)	5 (7)	2 (4)	28(31)	24 (25)	24 (12)	53 (14)
							
EPTB on LCC							
Treatment success	80 (35)	274 (49)	559 (52)	387 (36)	341 (34)	222 (43)	69 (50)
Default*	122 (52)	203 (36)	321 (30)	412 (39)	553 (56)	253 (49)	58 (42)
Death	2 (1)	3 (1)	3 (0.3)	7 (1)	8 (1)	14 (3)	-
Transferred out	28 (12)	85 (15)	198 (18)	260 (24)	91 (9)	24 (5)	11 (8)

* Patients with missing information on treatment outcome were assumed to have defaulted and analysed as defaulters.

PTB+ = smear positive pulmonary tuberculosis; PTB- = smear negative pulmonary tuberculosis; EPTB = extra-pulmonary tuberculosis

SCC = short course chemotherapy (2 months on RHZ+/- E or S, followed by 6 months on EH or RH)

LCC = long course chemotherapy (2 months on EH +/-S, followed by 10 months on EH; TH had also been used as an alternative to EH until its recent withdrawal).

R = Rifampicin,; H = Isoniazid; Z = Pyrazinamide; E = Ethambutol; S = Streptomycin; T = Thioacetazone.

When we adjusted the outcome measures for treatment results by various potentially confounding variables, significantly higher treatment success was exhibited among female patients, those 15–24 years, patients treated with SCC, those on re-treatment for relapse, PTB+ cases, those treated during 2000 and those treated in the Lemmo district health facilities (Table 7). Meanwhile, male patients, patients aged 45–54, PTB- cases, those on LCC, return after default cases and those treated in Duna district health facilities exhibited significantly higher default rate.

**Table 7:** Adjusted odds ratios for various factors that might affect treatment outcomes among registered tuberculosis patients

	**Treatment success (all TB)**			**Default (all TB)**		
	
Characteristic	Percent^1^	Adjusted OR* (95% CI)	p-value	Percent^1^	Adjusted OR* (95% CI)	p-value
**Sex**						
Male	45.5	1.00		37.5	1.00	
Female	49.4	1.15(1.08–1.23)	<0.001	34.0	0.88(0.82–0.94)	<0.001
**Age group (years)**						
0–14	42.6	0.83 (0.75–0.92)	<0.001	41.2	1.17 (1.06–1.30)	0.002
15–24 (reference group)	51.1	1.00		33.4	1.00	
25–34	47.5	0.84(0.77–0.91)	<0.001	34.4	1.07 (0.98–1.17)	0.14
35–44	45.7	0.76 (0.68–0.84)	<0.001	36.1	1.14 (1.03–1.28)	0.02
45–54	44.9	0.69 (0.60–0.79)	<0.001	37.7	1.26 (1.09–1.46)	0.002
55–64	51.4	1.04 (0.84–1.29)	0.71	35.4	1.04 (0.81–1.27)	0.90
≥ 65	37.4	0.67 (0.48–0.93)	0.02	43.7	1.27 (0.92–1.726	0.15
**Patient category**						
New	47.0	1.00		35.9	1.00	
Transferred-in	59.5	1.09 (0.89–1.36)	0.40	35.7	1.19 (0.95–1.48)	0.13
Return after default	33.9	0.68 (0.51–0.92)	0.01	52.9	1.57 (1.19–2.07)	0.002
Treatment failure	54.3	1.30 (0.91–1.86)	0.15	23.6	0.72 (0.48–1.09)	0.12
Relapse	62.9	1.51(1.01–2.26)	0.05	16.9	0.50 (0.30–0.82)	0.006
**TB type**						
Pulmonary positive	55.3	1.00		28.7	1.00	
Pulmonary negative	37.5	0.55 (0.50–0.60)	<0.001	43.7	2.06 (1.87–2.26)	<0.001
Extra-pulmonary	42.4	0.51 (0.47–0.55)	<0.001	41.1	1.94 (1.78–2.11)	<0.001
**Treatment regimen**						
Short course chemotherapy	55.5	1.00		27.7	1.00	
Long course chemotherapy	45.0	0.68 (0.62–0.75)	<0.001	40.9	1.46 (1.33–1.61)	<0.001
Treatment Centre**						
Hossana Hospital	27.0	1.00		40.0	1.00	
Lemmo district HF	60.4	5.14 (4.62–5.72)	<0.001	20.6	0.28 (0.25–0.31)	<0.001
Shashogo district HF	50.8	2.81 (2.41–3.28)	<0.001	44.1	1.18 (1.01–1.37)	0.04
Misha district HF	60.3	4.78 (4.23–5.39)	<0.001	33.0	0.58 (0.51–0.65)	<0.001
Gibe district HF	60.7	4.78 (4.20–5.45)	<0.001	26.2	0.51 (0.45–0.59)	<0.001
Soro district HF	50.5	2.70 (2.39–3.06)	<0.001	42.5	1.11 (0.98–1.26)	0.09
Duna district HF	30.2	0.77 (0.55–1.08)	0.12	67.3	5.18 (3.71–7.24)	<0.001
Badewacho district HF	41.1	1.64 (1.44–1.86)	<0.001	54.4	2.11 (1.86–2.40)	<0.001
**Year of treatment**						
1994	38.4	1.00		42.7	1.00	
1995	40.6	1.18 (0.99–1.39)	0.06	39.9	1.17 (1.00–1.37)	0.05
1996	48.7	1.29 (1.10–1.51)	0.002	33.3	0.83 (0.71–0.97)	0.02
1997	38.9	0.72 (0.61–0.85)	<0.001	39.5	1.08 (0.93–1.27)	0.32
1998	41.9	0.74 (0.63–0.87)	<0.001	43.8	1.20 (1.03–1.40)	0.02
1999	56.7	1.37 (1.16–1.62)	<0.001	30.0	0.59 (0.5–0.70)	<0.001
2000	59.8	1.41 (1.18–1.69)	<0.001	25.8	0.54 (0.45–0.65)	<0.001

OR = odds ratio; CI = confidence intervals; HF = Health facilities

* All the variables in the table are included in the model.

** All treatment centres in a district grouped together as a unit.

^1 ^Percentage out of total registered for treatment. Patients with missing outcome record were assumed to have defaulted and analysed as defaulters.

### Trend over time

DOTS was initiated in 1996 in a hospital and one health centre, with potential population coverage of 25% (defined as population living within 2 hours walking distance from a health facility; estimated at 250,000 for the hospital and 25000 for the health centre). The number of health facilities providing DOTS increased to 10 in 1997, 30 in 1999 and 41 in 2001, making the population coverage by DOTS 31%, 58% and 75% respectively. The proportion of patients treated with SCC increased from 7% in 1994 to 27% in 1998, 58% in 1999, and 97% in 2001. 95% (39/41) of the treatment centres had at least isoniazid, rifampicin, pyrazinamide and ethambutol at the time of visit for data collection. Reagents for Acid-fast stain were available in nine of the 13 diagnostic centres functioning at the time.

Simultaneously, treatment success for new PTB+ patients (on SCC and LCC together) increased from 38% in 1994 to 56% in 1998, 70% in 1999 and 73% in 2000 (χ^2^_trend_, p < 0.001). Defaulting among new PTB+ patients declined from 38% in 1994 to 30% in 1998, 20% in 1999 and 18% in 2000 (χ^2^_trend_, p < 0.001). Treatment failure decreased from 5% in 1994 to 1% in 2000. The proportion of reported deaths remained unchanged over years with some variations in the range of 2–5%.

### Treatment at small and large centres

Thirty-one out of 40 (77.5%) peripheral treatment centres had significantly higher treatment success, and 28 (70%) of them had significantly lower defaulter rate compared to the zonal hospital (details not shown). Further comparison by districts revealed that six of the seven rural districts had significantly better treatment success while three out of seven had lower defaulter rate than the zonal hospital (Table 7).

## Discussion

The results of our study show that there has been a continuous increase in treatment success and eventual decline in defaulter and failure rates in parallel to the expansion and decentralisation of DOTS to the lower treatment units. Treatment with SCC increased from a very low coverage in 1994 to almost full coverage in 2001 partly due to the expansion of DOTS (with SCC as one of its elements), and partly due to the regional TLCP commitment to SCC both in DOTS and non-DOTS areas. However, trends in case detection and notification showed inconsistencies over years.

In the hierarchy of study designs, observational studies come after the randomised controlled trials [17]. Nevertheless, in the real field condition, where randomised trials are not logistically feasible, such observational study designs may provide important and valid information. Our study design does not allow comparison of treatment outcomes between the DOTS and non-DOTS areas. However, this study gives us a valuable information on the program performance before and along the course of expanding DOTS in this resource-constrained rural setting.

Four years after the introduction of DOTS in the zone, it has been possible to achieve treatment success of 73% in 2000 for new PTB+ patients. Faring well towards the WHO/IUATLD recommended 85%, this confirms the finding of other studies [7,18] that the DOTS strategy works well in resource-constrained settings with low overall health coverage. Increased coverage by SCC, improved access to care through decentralisation of the service and improved patient follow-up with the introduction of DOTS have most likely played a significant role in improving the treatment outcomes.

Higher treatment success and lower default among female patients confirms previous study findings [11]. However, the proportion of females among patients registered for TB treatment was found to be consistently low, and it was exceptionally lower among patients older than 45 years. This may reflect a genuine gender difference in the TB epidemiology [19-21]. However, the possibility of this being a reflection of gender differentials in access to health care within the society may need to be ruled out by further studies.

Patients treated at the peripheral treatment centres exhibited better treatment outcomes compared to those treated in the zonal hospital, which implies better follow-up of cases or better access to the TB care services.

Despite significant decline in defaulter rate over time, patients that received re-treatment as "return after default", were much more likely to default again compared to new patients. This group of defaulters seems refractory to the conventional approach of treatment supervision; social and cultural factors that might play a role need to be explored. More death among patients on SCC was an indication of the policy that severely sick patients and those co-infected with HIV be a priority for treatment by SCC and these groups were more likely than others to die. Though lower among patients on SCC, the high proportion of failure among patients retreated for previous failure might signal the emergence of multi-drug resistant TB (MDR-TB). Earlier studies have shown that MDR has so far been below 1% [22,23].

The proportion of PTB+ was fairly stable in the range of 40–50% across the years, which indicates that the diagnosticians uniformly observed the diagnostic algorithms recommended by the NTLCP. The case detection rate (CDR), as estimated by the proportion of expected incident cases notified, showed an encouraging increase from 45% in 1994 to 115% in 1999, well above the CDR recommended by the WHO. The most plausible explanation for this might be that the vigorous implementation of the program during the earlier years of introduction of DOTS might have enabled detection of a big pool of prevalent cases of TB. But the pace in case detection could not be maintained during 2000 and 2001 when the CDR fell to 94% and 67% respectively. Reduction in the incidence of TB could be a possible explanation, but one cannot justify such a fast decline in a very short time. This calls for some case detection improvement initiatives to be in place as the proportion of TB cases detected and cured under DOTS is one of the health-related indicators of the millennium development goals [24]. Quality of the laboratory services and the possibility of under-reporting are among factors to be explored further.

Two major areas of program weakness need to be addressed. First, 94% of the patients were registered as new cases, which suggests that some re-treatment cases had been wrongly classified as new. Further, treatment outcomes have not been recorded for about one-fifth of the patients. Improving the record keeping system seems to be an immediate priority. Second, the proportion of PTB+ patients who had follow-up sputa examined for AFB was low. The threat of anti-TB drug resistance is imminent, and it is important to strengthen patient follow-up with sputum examinations. About one-third of the peripheral diagnostic facilities lacked reagents for Ziehl-Neelsen stain during the time of visit, and this might partly explain the low performance.

## Conclusion

The introduction and expansion of DOTS in Hadiya has led to a significant increase in treatment success and decrease in default and failure rates. The smaller institutions exhibited better treatment outcomes compared to the larger ones including the zonal hospital. The high number of patients with missing information in the unit registers is perhaps an issue that needs to be addressed as urgently as possible. Further studies are recommended to see the impact of the programme on the prevalence and incidence of tuberculosis.

## Competing interests

The author(s) declare that they have no competing interests.

## Authors' contributions

EBS was the principal investigator and participated in the design of the study, conducted the study, performed data entry and analysis, and wrote the manuscript. BL was the project co-ordinator and participated in the design, data analysis and write-up of the manuscript.

## Pre-publication history

The pre-publication history for this paper can be accessed here:


